# Artificial Intelligence Methodologies Applied to Technologies for Screening, Diagnosis and Care of the Diabetic Foot: A Narrative Review

**DOI:** 10.3390/bios12110985

**Published:** 2022-11-08

**Authors:** Gaetano Chemello, Benedetta Salvatori, Micaela Morettini, Andrea Tura

**Affiliations:** 1 CNR Institute of Neuroscience, Corso Stati Uniti 4, 35127 Padova, Italy; 2 Department of Information Engineering, Università Politecnica delle Marche, Via Brecce Bianche, 12, 60131 Ancona, Italy

**Keywords:** machine learning, neural network, deep learning, thermogram, skin resistance, plantar pressure, ulcer, lower limb wound, amputation, type 2 diabetes

## Abstract

Diabetic foot syndrome is a multifactorial pathology with at least three main etiological factors, i.e., peripheral neuropathy, peripheral arterial disease, and infection. In addition to complexity, another distinctive trait of diabetic foot syndrome is its insidiousness, due to a frequent lack of early symptoms. In recent years, it has become clear that the prevalence of diabetic foot syndrome is increasing, and it is among the diabetes complications with a stronger impact on patient’s quality of life. Considering the complex nature of this syndrome, artificial intelligence (AI) methodologies appear adequate to address aspects such as timely screening for the identification of the risk for foot ulcers (or, even worse, for amputation), based on appropriate sensor technologies. In this review, we summarize the main findings of the pertinent studies in the field, paying attention to both the AI-based methodological aspects and the main physiological/clinical study outcomes. The analyzed studies show that AI application to data derived by different technologies provides promising results, but in our opinion future studies may benefit from inclusion of quantitative measures based on simple sensors, which are still scarcely exploited.

## 1. Introduction

It was about two decades ago that international consensus emerged vigorously about the severity of diabetic foot syndrome among the complications of diabetes mellitus, leading to the first guidelines from an international working group [1]. In that report, it was indicated that among people suffering from diabetes worldwide, up to 10% develop foot ulceration in their life. An editorial on this consensus report highlighted that in the United Kingdom, about 9% of the National Health Service funds were spent on diabetes, and nearly half of them on hospitalization due to complications mainly related to the diabetic foot [1]. In addition to the healthcare costs, the personal costs were then emphasized in terms of poor quality of life, as well as the possible social costs. Indeed, patients with a diabetic foot often live with chronic ulcers, pain, and progressive deformity, and often undergo repeated drug medication (such as antibiotics) and outpatient attendances, as well as surgical procedures, with consequent enforced rest and time lost from work [1]. The clinical picture of course worsens dramatically when the diabetic foot pathology leads to amputation. Since the early 2000s, it has been well known that diabetic foot ulcers are the most common precursor of amputation and an important cause of morbidity and mortality in patients with diabetes [2,3]. In the United States, it was assessed that diabetic foot ulcers contribute to approximately 80% of the 120,000 non-traumatic amputations performed yearly [4]. Worldwide, it was reported that the global prevalence of diabetes mellitus was 425 million in 2017, with lifetime prevalence of the diabetic foot ulcers ranging from 19% to 34% [5]. Unfortunately, about one-third of such ulcers will not heal, eventually leading to some form of lower extremity amputation. In fact, every 20–30 s, a lower limb is lost due to diabetes somewhere in the world [5].

Is diabetic foot syndrome a multifactorial pathology? The answer is definitely yes. Indeed, diabetic foot pathophysiology includes at least three relevant etiological factors, i.e., peripheral neuropathy, peripheral arterial disease, and infection. Furthermore, trauma can be added, due to the use of inappropriate shoes or insoles [6]. In the majority of patients, peripheral neuropathy plays a central role (up to 50% of people with type 2 diabetes have neuropathy). Neuropathy leads to an insensitive and sometimes deformed foot, often with an impaired walking pattern that translates into abnormal biomechanical loading of the foot, subsequently leading to thickened skin (callus) and subcutaneous hemorrhage. In these conditions, even a minor trauma, caused by inappropriate shoes/insoles, or by an acute injury, can precipitate into a chronic ulcer [7]. Together with peripheral neuropathy, peripheral arterial disease is another major risk factor for diabetic foot syndrome. Peripheral arterial disease is specifically associated with impaired wound healing and lower extremity amputation. A small percentage of foot ulcers in patients with peripheral arterial disease are purely ischemic, and they are usually painful, but the majority of foot ulcers are either purely neuropathic or neuro-ischemic (that is, caused by combined neuropathy and ischemia), and they can often be asymptomatic [8]. Notably, the indicated factors can lead to foot ulcers of differing severity, possibly reaching a remarkable size and depth, as shown by images reported in several studies [9,10,11,12,13].

The illustrated situation indicates on one hand the complexity of diabetic foot syndrome, and on the other hand how insidious this syndrome can be, due to the frequent lack of early symptoms. This justifies the major attention received by diabetic foot syndrome in the latest years, also mirrored by the updates to the guidelines from the International Working Group on the Diabetic Foot, in 2015 and 2019 as well [14,15]. In this context, the importance of appropriate guidelines appears crucial, as it was reported that the implementation of a structured diabetes foot screening program could achieve up to 75% reduction in amputation rates [4]. Such a percentage of reduction is promising, but, on the other hand, it indicates that the problem of diabetic foot-related amputations is far from being solved. Further effort therefore needs to be carried out.

We have briefly summarized the complexity of diabetic foot syndrome. In such complex pathophysiological problems, significant benefits can nowadays be provided by artificial intelligence for the smart analysis of data derived from different sensors and technologies, of relevance for screening, diagnosis and care of the diabetic foot. In fact, artificial intelligence has unique capabilities to analyze problems affected by the behavior or the condition of a wide battery of factors and measured parameters, finally providing crucial indications about those that are more relevant to focus on in the problem under investigation. In the general field of diabetes, artificial intelligence has proven to be effective for several applications, as summarized by many review studies, such as those by Fregoso-Aparicio et al., Nomura et al., Tan et al., and Gautier et al., to mention some of the latest [16,17,18,19]. However, studies related to diabetic foot syndrome, with exploitation of the artificial intelligence applied to different technologies, are not as common as for other diabetic complications, such as diabetic retinopathy [20]. On the other hand, it is worth noting that a not negligible number of studies on artificial intelligence in diabetic foot are emerging, especially in very recent years (from 2020 onwards). In fact, considering artificial intelligence’s capability in addressing complex problems, and, as previously illustrated, diabetic foot syndrome being such a problem, we definitely expect further studies in the field. In this review analysis, we aimed to summarize those studies carried out so far, and draw some conclusions about possible future directions of research. Specifically, this review study is intended as a sort of guide for those investigators aiming to develop new and improved methods for diabetic foot data analysis, and for those users aiming to establishing among the already proposed methods the most appropriate for application to their data.

## 2. Scientific Literature Search Strategy

The search through the scientific literature was performed in PubMed by one of the study authors, then checked and agreed upon by another author.

Following testing of different PubMed search strings, we identified this final string:

diabet*[ti] AND (foot*[ti] OR feet*[ti] OR ulcer*[ti] OR skin*[ti]) AND (((machine*[tw] OR deep*[tw]) AND learning*[tw]) OR (artificial*[tw] AND intelligen*[tw]) OR (data*[tw] AND mining*[tw] OR (neural*[tw] AND network*[tw])))

According to PubMed guidelines, “ti” searches in the article title, whereas “tw” (“text word”) enables searching in all main fields of PubMed records, i.e., in the title, abstract, MeSH terms, plus some additional fields. The symbol “*” enables searching for all variations of a word root: e.g., intelligence, intelligent, etc. Notably, we used different terms for our search, which are sometimes used interchangeably, though strictly speaking they are not. Specifically, in this literature search, the pair of terms occurring most frequently was “machine learning”, which indicates a series of techniques that are part of the more general artificial intelligence framework (though, to our knowledge, machine learning identifies a wider portion of artificial intelligence in data analysis).

**Figure 1:**
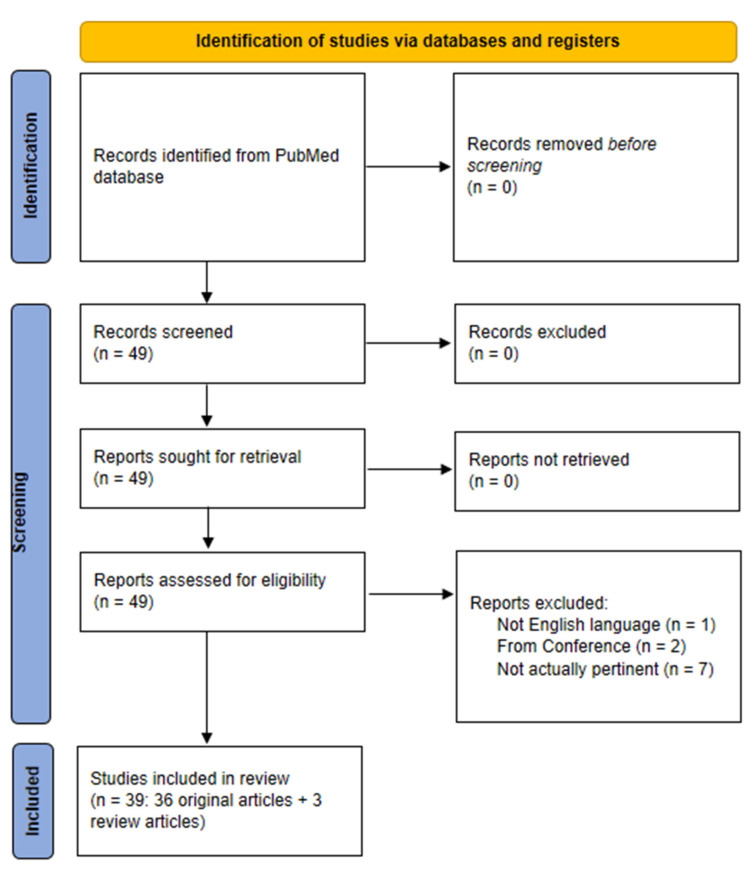
PRISMA flow diagram of the literature search strategy.

The indicated search strategy yielded 49 items (last check: 20 September 2022). We therefore analyzed each item, and ended with a set of 36 articles pertinent for our analysis (plus three review studies that will be indicated in the discussion section). The PRISMA flow chart of our literature search is reported in Figure 1.

In addition, from the reference list of the selected articles, we identified another 4 pertinent articles which were not captured by our search strategy, thus for a total of 40 articles included in the review. Interestingly, 33 out of these 40 articles were published from 2020 onwards, indicating rapidly expanding interest in the investigated topic. In this review, we did not consider articles from conference proceedings, or those not in English language.

In the following sections, we summarize the main aspects of the selected studies, with focus on both the main physiological/clinical study goals and outcomes and the methodological aspects related to artificial intelligence/machine learning techniques. Articles are presented in two separate sections: the first section is related to studies concerning the screening for diabetic foot syndrome and the assessment of risk for diabetic foot ulcers, whereas the second section describes studies related to the identification of already present diabetic foot ulcers, of the lesion severity and possible treatments. Each section is then divided into two subsections, one related to the exploitation of different clinical, socioeconomic and sociodemographic data as inputs for the machine learning algorithms, and the other characterized by the inclusion of image-based data. In each subsection, articles are reported in chronological order. 

## 3. Artificial Intelligence in Diabetic Foot Syndrome: Methodological Approaches and the Main Physiological and Clinical Outcomes

### 3.1. Screening for Diabetic Foot Syndrome and Risk Prediction for Ulceration

#### 3.1.1. Screening and Risk Prediction: From Clinical, Socioeconomic, Sociodemographic Data

Summary information about the studies presented in this section is reported in Table 1.

The first study pertinent to our review was dated 2013 [21]. In that “pioneering” study, Singh et al. aimed at analyzing patients with type 2 diabetes mellitus (T2DM) to find the risk for a diabetic foot ulcer (DFU), in relation to five single nucleotide polymorphisms (SNPs) in the TLR4 gene (namely, Asp299Gly (rs4986790), Thr399Ile (rs4986791), rs11536858, rs1927911, and rs1927914), by using an artificial neural network (ANN). A total of 255 T2DM individuals, namely 125 patients already with DFU and 130 patients without DFU assumed as the control group, were enrolled in the study. All participants underwent clinical and laboratory evaluation, and family history, life habits and duration of disease were recorded through a questionnaire. Genomic DNA was extracted from peripheral blood and the SNPs were analyzed to find allelic combinations that may alter the risk of DFU. The final ANN architecture consisted of 5 input nodes (the SNPs), 10 hidden layer nodes, and 1 output node representing the risk of DFU. For validation purposes, with the same set of data used for training and testing, a conventional statistical multivariate linear regression (MLR) was also carried out. The ANN model with the five SNPs as inputs was able to correctly predict 83% of the validation set (25% of the dataset), i.e., the presence or absence of DFU. The conventional statistical MLR model correctly predicted 74% of the cases only. Based on the study findings, it was concluded that some haplotypes of the analyzed SNPs may be involved in the pathogenesis and progression of DFU, whereas, in contrast, some others appear to be protective against DFU.

Since 2020, several studies have been published that are pertinent for our review. The work by Ferreira et al. aimed at using an unsupervised learning technique to automatically classify the risk of developing diabetic foot syndrome before any visual change can be perceived [22]. The developed method did not require clinical exams, physical contact with the patients or foot imaging. It was based on a questionnaire collecting data related to the health conditions of the patients and to the changes they had felt in their feet, such as numbness, loss of sensation, and tingling. Additionally, information on daily foot care, on the type of footwear commonly used, and on the patient’s socioeconomic and sociodemographic conditions was collected. For each of the 239 T2DM patients, the database included a record containing 54 variables. After normalization, a competitive neural layer (CNL) was trained to cluster the data into two groups by using the Kohonen Learning Rule. A nurse specialized in diabetic foot diagnosis and prevention verified the presence of variables considered to be risk factors for the development of the condition. Her classification was assumed as the ground truth. Then, the CNL-based architecture performed its selection and hence clustered the patients into two groups, i.e., Group A with 127 patients, and Group B with 112 patients, the latter representing patients having the greatest risk of developing diabetic foot. Out of 54 variables, the 15 variables presenting absolute weight greater than 0.5 were selected (age, type of diabetes, body mass index (BMI), food control, physical activity, smoking, hypertension, circulatory problems, sensation of shock in feet and legs, presence of bunion, visual changes, habit of washing the feet, presence of calluses, presence of wound, and presence of amputation). The model was applied to the randomly selected test set composed of data from 73 subjects, reaching sensitivity of 71%, specificity of 100%, and accuracy of 90%. Following comparison with previous relevant works, the authors concluded that the proposed method has the advantage of non-invasiveness, and it does not require image processing or sensors to monitor the patient’s feet. On the other hand, it is obvious that such a method, also based on subjective information provided by the participants, may provide in some cases unreliable predictions.

The study by Schäfer et al. aimed at understanding whether different sources of patient’s health and socioeconomic data (collected at not specific time intervals in a period of almost twenty years) can be leveraged through machine learning techniques to assess the risk of developing DFU, as well as subsequent amputation [23]. Using available socioeconomic registry data and the medical records of 246,705 patients with diabetes, the study was organized into two steps. First, through survival analysis, it was investigated what risk factors are associated with DFU or amputation. After identifying the risk factors, machine learning models were applied to predict the occurrence of DFU/amputation at different time intervals across two different setups: in the first one, the aim was classifying whether patients develop DFU/amputation based on their medical history up to a maximum of *n* years, with *n* ranging from 3 to 11. The second setup considered patient’s information in a prescribed time interval after diabetes onset (from a minimum of 3 to a maximum of 11 years) and predicted the development of DFU/amputation within the next 2, 3 or 5 years. Logistic regression (LR) and random forest (RF) classifiers were used. It was shown that important risk factors for DFU/amputation are low family income, cardiovascular and chronic renal complications, peripheral artery disease, and neuropathy. By evaluating receiver operating characteristic (ROC) curves obtained from the classifiers, it was concluded that in the first setup, both classifiers were able to distinguish between patients with and without DFU/amputation. In contrast, the second setup did not show sufficiently accurate prediction, thus suggesting the need for improvements in the implementation of the predictive models.

In 2021, Stefanopoulos et al. published a study based on a conditional inference tree (CTREE) algorithm for the prediction of DFU risk in an inpatient population [24]. Nationwide Inpatient Sample datasets (USA) from 2008 to 2014, including over 10 million diabetic patients (of which 326,853 had DFU), were used for model generation and testing. The CTREE classifier, i.e., a decision tree that estimates relationships by means of binary recursive partitioning, was used to predict the incidence of DFU. An initial selection was performed to identify potential predictors. The chi-square test was used to identify categorical variables with different frequency values between subjects with and without DFU (those variables therefore being potential significant predictors for the output). Other appropriate tests were performed on continuous variables for the initial selection (maintaining only those with significant regression with the output, i.e., the presence or absence of DFU). Subsequently, a LASSO (Least Absolute Shrinkage and Selection Operator) regression was performed for the final selection of predictors, yielding six predictors. In addition, a different strategy was applied for predictor selection, based on backward and forward regression, in this case yielding 10 predictors. Finally, CTREE was applied on both the 6- and 10-predictor sets to derive optimal cut-off values for each predictor. Performance was assessed by accuracy, sensitivity, specificity and AUC_ROC_, i.e., Area-Under-the-Curve of the ROC curve. The 6- and 10-predictor models achieved similar performance, and thus the more parsimonious 6-predictor model was considered preferable.

In 2022, the study by Haque et al. aimed to predict diabetic neuropathy (a risk factor for DFU) or already overt DFU by analyzing some biomechanical variables, specifically muscle electromyography (EMG) parameters and ground reaction forces (GRF) [25]. The analyzed dataset included 21 subjects, with diabetic neuropathy (*n* = 6), with diabetic neuropathy already complicated by ulceration (*n* = 9), and with neither diabetic neuropathy nor ulceration, assumed as control subjects (*n* = 6). The EMG data were derived from the right vastus lateralis, gastrocnemius lateral, and tibialis anterior, whereas the ground reaction force data included three-dimensional components (GRFx, GRFy, GRFz). All data underwent appropriate processing to derive several parameters (features for machine learning analysis), especially from the EMG signal, including the mean absolute value, slope changes, number of zero crossings, skewness, signal moment. High-correlation feature elimination was then performed to optimize the subsequent feature ranking phase. The correlation matrix between pairs of features was calculated using pairwise linear correlation, and in the case of values higher than 0.9, one of the two features in the pair was discarded. Thereafter, four feature selection approaches were used for feature ranking (chi-square, Minimum Redundant Maximum Relevant, Neighborhood Component Analysis, and the ReliefF algorithm). For the classification in the control, diabetic neuropathy or DFU condition, eight different algorithms were trained—that is, Discriminant Analysis (DA), Ensemble Classification (EC), Kernel Classification (KC), K-Nearest Neighbor (KNN), Linear Classification (LC), Naïve Bayes (NB), Support Vector Machine (SVM), and Binary Decision Classification tree (BDC). The best-performing algorithm was KNN. This algorithm was thus optimized to tune the hyperparameters by using Bayesian optimization. The best accuracy reached 96.18%. Other performance metrics were AUC, sensitivity (recall), precision, and F1-score, i.e., a harmonic average of sensitivity and precision.

In the study by Nanda et al. [26], a model was developed for predicting the onset of DFU and its grading according to the Wagner score (five classes). Eighty patients with T2DM and with DFU, as well as eighty patients without DFU, were enrolled. Clinical and laboratory biochemical risk factors were considered as features for different machine learning algorithms (up to 32 features, among which 23 were continuous and 9 were categorical). More precisely, the features list included diabetes duration, insulin use, ulceration duration, Wagner ulcer classification, the presence of diabetic complications, smoking history, blood/plasma parameters, as well as sex and anthropometric parameters. To gain insight into the importance of the different features, four different feature ranking algorithms were used (ReliefF, Info Gain, Gain Ratio and chi-squared). The analyzed machine learning algorithms were SVM (with Poly kernel and RBF (Radial Basis Function) kernel), NB, KNN, RF and three ensemble learners (Stacking C, Bagging and AdaBoost). These models were exploited first to discriminate between the two groups of patients (stage I classification), and secondly for ulcer grading classification (stage II classification). In stage II, since the five DFU classes had different numbers of samples, the Synthetic Minority Oversampling Technique (SMOTE) was used to obtain a balanced dataset. The top features predicting DFU were interleukin-10 (IL-10), fasting plasma glucose, alipoprotein A1 (Apo A1), neuropathy presence, low-density lipoprotein and triglycerides. As regards stage II, diastolic blood pressure, uric acid, postprandial plasma glucose, sex, low-density lipoprotein and IL-10 were the top features discriminating between different ulcer grades. In terms of the best machine learning approach, the ensemble learning algorithms performed better than individual classifiers, according to various performance metrics (Matthews correlation coefficient (MCC), which is an appropriate formula of true/false positive/negative values, as well as F1-score and AUC_ROC_).

In the study by Troitskaya et al., some polymorphisms of genes related to vascular tone regulation factors, platelet receptors, vascular wall remodeling and prothrombotic factors were studied, as well as some markers of endothelial dysfunction, such as MMP9 (metalloproteinase-9) [27]. The hypothesis was that these different biomarkers may help to predict the possible development of diabetic foot syndrome, considering the multifactorial nature of the syndrome. For this purpose, 397 patients with diabetes were studied, of which 198 had no complications and 199 demonstrated some signs of diabetic foot syndrome. By means of neural network analysis, with the Multilayer Perceptron (MLP) approach, it was found that the indicated data allow for predicting the development of diabetic foot syndrome with an accuracy of 92.9%. Other considered metrics were sensitivity, specificity and AUC_ROC_. However, no details were provided about the MLP approach.

#### 3.1.2. Screening and Risk Prediction: From Imaging

Summary information about the studies of this section is reported in Table 2.

In 2018, Toledo Peral et al. developed an application for skin macule characterization, based on a three-stage segmentation and characterization algorithm [28]. Specifically, the purpose was to classify vascular macules, petechiae, and macules due to trophic changes or trauma from photographs of the lower limbs of diabetic patients. Indeed, it was claimed that such diabetic skin manifestations are among the first symptoms of vascular damage, and may precede the onset of DFU. A group of 19 diabetic patients was studied, with 82 skin macules, but no DFU. For the study purposes, the first step was the acquisition of color photographs of the skin macules from the lower limb (stage 1), then images segmentation was performed to identify the skin regions with macules (stage 2), and finally the macules were characterized in terms of the severity of the skin damage. Thereafter, the macule features were fed into an ANN classifier (feedforward backpropagation architecture, two hidden layers and four neurons per layer), which demonstrated a 97.5% accuracy in differentiating between the different macule types.

In 2020, the study by Cruz-Vega et al. analyzed the use of machine learning and deep learning techniques for the classification of diabetic foot thermograms [29]. Different approaches were compared: two traditional machine learning classifiers (MLP and SVM), two models based on pre-trained convolutional neural networks (CNN), such as GoogLeNet and AlexNet, and a new CNN proposed by the authors, i.e., Diabetic Foot Thermograms Network (DFTNet). All methods were applied to 110 thermograms of patients with diabetes, obtained from a public thermogram database. For the selection of the regions of interests (ROIs), required for the use of the MLP and SVM algorithms, a histogram-based segmentation method was exploited, obtained by fuzzy logic approach according to the measure of entropy. The CNN classifiers required additional images to prevent the network from overfitting. For this reason, each image was divided into patches, increasing the dataset by about ten-fold. The foot was divided into four angiosomes, i.e., the medial plantar artery, lateral plantar artery, medial calcaneal artery and lateral calcaneal artery. A thermal change index was used to measure the difference between corresponding angiosomes from each subject and related reference values were properly established. The algorithms were trained to classify five classes of thermal changes in the plantar regions. The pre-trained CNN AlexNet was fine-tuned by replacing some layers. Little information was reported on the GoogLeNet network, but it appears that GoogLeNet was exploited for the development of the new network. Indeed, in DFTNet, the number of layers was reduced to 9, starting from the 22 layers of GoogLeNet, this allowing a decrease in the training time. The results showed that the best two algorithms were DFTNet and MLP, with AUC_ROC_ of 0.8533 and 0.8333, respectively. Other performance metrics were sensitivity, specificity, precision, accuracy, and F1-score. It was claimed that possible future works include increasing the number of thermograms, improving the structure of DFTNet, and reducing the need for participation of human experts in the selection of patches and ROIs.

In 2021, the study by Khandakar et al. was somewhat similar to that by Cruz-Vega et al. [29] performed one year earlier, as both studies shared the exploitation of thermograms for the early detection of diabetic foot abnormalities (and hence for possible assessment of DFU risk) [30]. In Khandakar’s study, artificial intelligence models were trained on a dataset related to 122 patients with diabetes and 45 control subjects, including for each subject gender, age, weight, height, and foot pair thermograms. In this study, the purpose was simply to distinguish between diabetic and control subjects (i.e., two-class classifier). Machine learning algorithms were applied to features extracted from the thermograms, whereas deep CNN algorithms were applied to the whole images. Regarding the feature-based detection problem, careful feature selection was carried out to reduce the possibility of overfitting: redundant features were removed, and machine learning algorithms were applied to obtain different feature ranking sets. Feature ranking was performed by the Multi-Tree Extreme Gradient Boost (XGBoost), RF, and Extra Tree techniques. Different feature subset combinations were then tested as predictors. The implemented classifiers were MLP, XGBoost, LR, Adaptive Boosting (AdaBoost), KNN, SVM, Extra Tree, RF, Gradient Boosting and Linear Discriminant Analysis (LDA). Regarding the deep CNN models, transfer learning (i.e., use of pre-trained models) from ImageNet database (with more than one million images) and image enhancement techniques were applied to cope with the small size of the dataset. The implemented CNN algorithms were ResNet18, ResNet50, DenseNet201, InceptionV3, MobileNetV2, and one Visual Geometry Group (VGG) network, specifically VGG19. To avoid an imbalanced training dataset and consequent possible biased estimates, SMOTE was used for training data augmentation. The 10-feature AdaBoost algorithm was found to be the best classifier (performance metrics were sensitivity, specificity, precision, AUC_ROC_, F1-score). Finally, the inference time of the algorithms was also assessed, this being relevant to consider the opportunity for implementation of these algorithms on smartphones, allowing early DFU risk detection even in the patient’s home setting.

In the same year, the study by Arteaga-Marrero et al. employed deep learning techniques, compared with more conventional techniques, to obtain a proof of concept for foot sole segmentation of multimodal images (consisting of visual-light, infrared, and depth images), providing spatial information of the sole [31]. Indeed, the authors claimed that the lack of standardized foot sole segmentation is one of the greatest technical obstacles for the inclusion in standard care protocols of the thermography technique, which is important for DFU prevention. The proposed approaches for automatic segmentation included conventional skin segmentation not exploiting artificial intelligence algorithms, a CNN (U-net, previously generated and trained by the authors), and a deep CNN (SegNet) for semantic pixel-wise segmentation. The different techniques were compared based on performances over a dataset of 74 images by 37 healthy subjects. Manual segmentation was used as ground truth to evaluate performance, expressed in terms of spatial overlap, accuracy and precision. The conventional approach and the U-net approach performed best, at least in terms of spatial overlap. The other considered performance metrics were sensitivity, specificity, and Dice Similarity Coefficient (DICE). It was concluded that automatic foot sole segmentation could be used to substitute the time-consuming manual segmentation approach.

The study by Dremin et al. was not specifically focused on the diabetic foot issue, but more generally on skin abnormalities, which in diabetic patients can lead to complications such as DFU [32]. Specifically, the study addressed the problem of protein glycation, which causes dysfunction of tissues containing collagen. In fact, the structural and functional changes in collagen contribute to the development of alterations affecting the skin, blood vessels, and nerves, fostering the onset of different pathologies, such as DFU. In the study, photonics-based technology was exploited to derive biomarkers of protein glycation. In more detail, polarization-sensitive hyperspectral imaging and parameters allowed the calculation of distribution maps for skin blood content and blood oxygenation, and of the polarization index of reflected radiation. These biomarkers were proven able to differentiate the skin condition, as well as the microcirculation state, between diabetic and healthy subjects. The subjects analyzed were 32 healthy subjects in a first study phase, and a group of 20 diabetic patients plus a further 20 healthy subjects in the second study phase. The machine learning approach used to analyze the data was an ANN, of MLP type, with an input layer, one hidden layer, and a linear output layer. Performance metrics were AUC_ROC_, sensitivity and specificity.

In 2022, Khandakar et al. continued the previous research [30] on thermograms analysis [33]. As a fact, the methodological approaches were very similar to those of the previous study [30]. In this new study, it was concluded that one of the analyzed classifiers (the MLP one), applied to the features extracted from the thermograms, showed an accuracy that outperformed those reported in the literature over the same dataset. In a second study of the same year [34], Khandakar et al. also focused on the aim of clustering the thermograms based on the severity of the abnormalities in the temperature patterns. From the methodological point of view, the novelty of this study was the exploitation of the K-mean clustering technique for unsupervised cluster identification.

In the study by Zhang et al., the aim was to predict the onset of DFU, and of its severity, by lower extremity computed tomography angiography (CTA), complemented by other clinical data, and sociodemographic data [35]. A group of 203 patients with possible diabetic foot syndrome were analyzed, and divided into two subgroups based on the severity of the DFU according to an appropriate grading index (Wagner score), ranging from grade 0 (no skin lesion) to grade 5 (gangrene of the entire foot). According to a specific cut-off, 138 patients were assigned to the low Wagner score group, and 65 patients to the high Wagner score group. Based on CTA data, 10 predictive features were selected for inclusion in the model. The total dataset was randomly split into training, testing and holdout samples (3:1:1 ratio). An ANN model was created, based on the MLP algorithm. The MLP model was composed of three layers (input layer, hidden layer, and output layer). The model used predictive factors from the input layer (age, gender, BMI, duration of diabetes, duration of a diabetic foot ulcer, limb symptoms, degree of lower-extremity arterial stenosis, segment of lower-extremity arterial stenosis, arterial calcification, and comorbidities) and the output layer (low or high Wagner score). A LR model was also developed as a control for the MLP model. The metrics considered for the model performance over the holdout sample were accuracy, sensitivity, specificity, positive predictive value (PPV), and negative predictive value (NPV), which were 88.9%, 90.0%, 88.5%, 75.0% and 95.8%, respectively. The AUC_ROC_ was also considered (0.955), which was remarkably superior to that of the classic LR model. It was concluded that the MLP model could accurately predict the onset and severity of a DFU.

In the study by Bouallal et al., the main aim was to develop an automated accurate algorithm for the segmentation of the diabetic foot [36], with the consideration that appropriate segmentation is important for the interpretation of thermal images, which can predict the onset of DFU. A dataset consisting of 398 pairs of thermal and RGB (Red, Green, Blue) images was studied, obtained from 145 diabetic patients and 54 healthy subjects. A deep neural network architecture was proposed, named Double Encoder-ResUnet (DE-ResUnet), which includes encoder and decoder pathways (starting from the thermal image and ending with the segmentation output), with skip connections between the corresponding layers. The proposed new network combines the advantages of U-Net and ResNet architecture. The former belongs to the category of fully convolutional networks, allowing data augmentation and facilitating the propagation of information among the different network layers. The latter is a residual network, able to propagate the information with minimum degradation thanks to the skip connections, and allowing accurate object detection and semantic segmentation, i.e., appropriate labelling of specific image regions. Moreover, the proposed network fuses thermal and color information to improve segmentation accuracy. This approach was found to be able to accurately delineate the regions of toes and heels that are at high risk for ulceration, and outperformed other methods, reaching an average intersection over union (IoU) of 97%. The accuracy per class (Acc) metric was also considered.

The study by Muralidhara et al. [37] was similar to those of Khandakar et al. [30,33,34], as it exploited the same dataset (122 diabetic patients, and 45 control subjects). Two levels of multi-class classification were considered, namely a five-class classification of the diabetic thermograms and a six-class classification including the non-diabetic thermograms as an additional class. This consideration of diabetic samples of different grades along with non-diabetic thermograms (defined as “holistic classification”) provides additional relevant information during the training process, resulting in a more robust classifier. The problem of class balancing was addressed by weighted classification (adjusting class weights to assign higher importance to minority classes) and by data augmentation (generating multiple slightly different versions of images through image rotation, scaling, flipping, and cropping). Then, a CNN was proposed for discrimination between non-diabetes (i.e., no DFU) and the five severity DFU grades according to the thermal images, and the new network performance was compared to those of pre-trained networks such as AlexNet. The considered metrics were accuracy, specificity, sensitivity, precision, and F1-score. The achieved accuracy was 0.9827.

### 3.2. Overt Diabetic Foot Ulcer Detection, Grading, Prognosis and Care

#### 3.2.1. Overt Diabetic Foot Ulcer Focus: From Clinical, Socioeconomic, Sociodemographic Data

Summary information for this section is reported in Table 3.

The first study that we include in this section of our review is that by Yusuf et al. in 2015, which focused on the use of an electronic nose (e-nose) for the detection of single and multiple pathogens responsible for infections in the ulcerated diabetic foot [38]. Indeed, the response of the e-nose is known as a “smell print”, and different bacteria may exhibit different smell print patterns. In more details, the work aimed to determine a proof of concept for the use of the e-nose in the identification of microbial species in vitro, to subsequently move on to its use in the clinical context. Data were obtained by media culture preparation and bacteria isolation, retrieved from samples of diabetic foot wounds (wild-type bacteria) and by the American Type Culture Collection (standard bacteria). The e-nose was applied on the resulting dataset, i.e., the agar medium with the bacteria, and different machine learning multi-class odor classifiers were tested to evaluate the ability of the e-nose in identifying single/multiple pathogens. The used classifiers were SVM, KNN, LDA, and Probability Neural Network (PNN). These four classifiers were implemented via the leave-one-out cross-validation technique, using two different sets of predictors: the first set consisted of the features retrieved by principal component analysis (PCA), and the second set was obtained by also applying LDA to the first set, to reduce the high dimensionality of the feature space. Performances of the classifiers were evaluated in terms of accuracy, precision, specificity, and sensitivity. Almost all of the classifiers achieved a precision of at least 90% and an accuracy of at least 89%.

In 2018, Huang et al. exploited a Radial Basis Function Neural Network (RBFNN) to quantify the rehabilitative efficiency of the Buerger’s exercise, which is a rehabilitation technique that improves blood flow in the lower limbs, possibly leading to a reduction in the risk of amputation due to DFU [39]. The RBFNN classifier was used to discriminate between healthy and diabetic subjects, based on tissue oxygen saturation in lower limbs and relative total hemoglobin concentration as model predictors, which are measured at different locations of the foot. The dataset included 30 diabetic and 15 healthy subjects. In the RBFNN model, radial basis functions were used as activation functions. The model output was a continuous value between 0 and 1, with these limits corresponding to healthy and diabetic subjects, respectively. An optimal cut-off for the output value was searched to distinguish between the two participants’ categories. However, the continuous output value was itself an informative index, quantitatively assessing the state of blood circulation. If this index is computed before and after Buerger’s exercise, its variation can be assumed as a quantitative index of rehabilitative efficiency. The neural network performance was evaluated using the F1-score classification metric. It was concluded that the neural network performed satisfactorily, achieving an F1-score of 80%.

In 2020, the study by Lin et al. aimed at generating models for the prediction of amputation/mortality of patients with DFU [40]. Three predictive models were generated: a traditional Cox regression model, and two models from the artificial intelligence framework, i.e., a Back-Propagation Neural Network (BPNN) and a BPNN based on genetic algorithm (GA) optimization. Each of the three models was implemented with two different setups. In one setup, the goal was to predict whether the patient would have been subject to amputation; in the second setup, the model tried to predict whether the subject would survive for three years. Based on biochemical indicators (blood/plasma parameters) selected through cluster analysis, combined with clinical data and the presence of complications (related to 200 patients), the Cox regression model was implemented first. In the BPNN model (the “basic” one), three risk factors, identified by the Cox regression model, were used as initial predictors. Finally, the BPNN based on GA was built upon the basic BPNN, allowing model optimization by the simulation of Darwin’s theory of the process of biological evolution, and the process of the biological evolution of genetic mechanisms. Analyses were performed separately for the prediction of amputation and prediction of mortality. Performances were assessed through the AUC_ROC_, sensitivity and specificity metrics. The predictive models based on optimized BPNN and basic BPNN were superior to the model based on simple Cox regression analysis, both for amputation and mortality prediction. However, the study failed to demonstrate improved performances of GA-based BPNN compared to basic BPNN.

In 2021, the study by Du et al. explored characteristics of inpatients suffering from DFU before (2019) and after lockdown (2020), due to the COVID-19 pandemic [41]. The study searched for common features associated with amputation/mortality in patients with DFU, and subsequently generated predictive models of the indicated outcomes. Exploited data included clinical and laboratory values, and Wagner ulcer classification and Wound, Ischemia, and Foot Infection (WIFI) classification. A group of 23 patients were studied. Based on the available data, authors identified a longer delay in admission and a higher risk of mortality in subjects suffering from DFU in the post-lockdown period. Six machine learning prediction models for amputation/mortality were developed to identify risk factors: LR, SVM, RF, gradient boosting decision tree (GBDT), ANN, and XGBoost. For hyperparameter tuning, three-fold cross-validation was used in each model. Performances were assessed using AUC_ROC_, accuracy, sensitivity, specificity, PPV, and NPV. The XGBoost model outperformed the others in terms of AUC_ROC_, accuracy, sensitivity, and NPV. As regards amputation, the main risk factors were white blood cell and blood potassium levels, and pre-hospitalization in the pre-lockdown period, whereas pre-hospitalization, foot ischemia and serum albumin levels were identified as risk factors in the post-lockdown period. For mortality, the main risk factors were age, and both foot and non-foot infections. It was concluded that DFU patients with any kind of infection should require prompt intervention.

In 2022, the study by Xie et al. aimed at developing an accurate prediction model that could estimate the probability of in-hospital non-amputation, minor amputation (i.e., amputation below the ankle) or major amputation in patients with DFU, providing individualized analyses of the patients’ risk factors [42]. Light Gradient Boosting Machine (LightGBM) with five-fold cross-validation was used to develop a multi-class classification model, which incorporated 37 baseline characteristics of 618 patients. Bayesian hyperparameter optimization based on the Tree–Parzen estimator was used to determine the optimal calibration set under the cross-validation procedure. This set was used to calibrate the model predictions based on isotonic regression, while a specific score (Brier score) was used to evaluate the coherence between predicted and observed probabilities. Among the participants’ data, the collected data were demographic characteristics, medical and medication history, clinical and laboratory data, and WIFI classification. The model performance was evaluated using five evaluation metrics (AUC_ROC_, sensitivity, specificity, PPV and NPV). The overall model performance was the weighted average of the performance in each category. It was shown that the proposed multi-class classification model had strong predictive power, with high weighted-average AUC_ROC_ (0.90), and acceptable sensitivity (87.1%), specificity (74.4%), NPV (79.7%) and PPV (86.3%). In addition, to provide a visual interpretation of the contribution of each patient’s characteristics to the model predictions, the Shapley Additive explanations (SHAP) algorithm was used. Precisely, SHAP values provided a direct measure of the influence of each patient’s variable on the actual predictions under the interaction with other variables. In the authors’ view, the SHAP algorithm, enhancing the transparency of the model, could promote its acceptance by physicians. The study was, however, conducted retrospectively, and missed external validation cohorts.

In the study by Margolis et al., the main aim was to demonstrate by a machine learning approach that simple DFU characteristics, such as wound area and wound duration, can predict wound healing [43]. For this purpose, a multicenter study, called the Diabetic Foot Ulcer Consortium (DFUC), was carried out, including 204 patients with DFU. The main study outcome was a healed wound by the 16th week of care. LR and LASSO were exploited to build a prediction model of the outcome. Features considered for the model development were wound area, wound duration, wound depth, wound site (categorical variable), arterial flow, patient’s BMI, and history of dialysis. The performance of different models was assessed by the AUC_ROC_. It was found that wound area and duration were the most relevant predictors of wound healing, their combination providing AUC_ROC_ of 0.71. The other features were shown to add little to that AUC_ROC_ value.

The premise of the study by Deng et al. was that severe infections, including an acute DFU infection, can induce an acute hyperglycemic crisis episode (HCE), such as diabetic ketoacidosis and hyperglycemic hyperosmolar status [44]. Thus, the study aimed to investigate risk factors for mortality in patients with DFUs and HCE. A total of 27 inpatients with DFUs concomitant with HCE were compared to 93 inpatients with isolated DFUs. Amputation and survival rates were compared over a 6-year period. XGBoost was used to explore the relative importance of HCE (and other risk factors) to all-cause mortality in DFU patients. It was found that HCE is a major risk factor for mortality in patients with DFUs, whereas no difference was observed between DFU+HCE and isolated DFU groups as regards the amputation rate. Performance metrics for the XGBoost model were AUC_ROC_, accuracy, sensitivity, specificity.

#### 3.2.2. Overt Diabetic Foot Ulcer Focus: From Imaging

Summary information for this section is reported in Table 4.

In 2017, Wang et al. carried out a study aimed at the identification of the wound area of DFUs, since the wound area (more precisely, its possible reduction over time) was assumed as a relevant indicator of successful wound healing process [45]. For this purpose, after the segmentation of images in some regions (superpixels), a cascaded two-stage classifier was used. In the first stage, a set of binary SVM classifiers were trained and applied to different subsets of the entire training images dataset, and incorrectly classified instances were collected. In the second stage, another binary SVM classifier was trained on the incorrectly classified set. Various color and texture descriptors were extracted from the image superpixels and used as the input for each stage in the classifier training. Finally, the detected wound boundary was refined by applying conditional random field methods. A group of 15 patients were tracked over a two-year period, providing 100 high-resolution DFU images. The results showed that the proposed approach was superior to other classifiers that were analyzed (specifically, single-stage SVM-based classifier with the same configuration of the first-stage classifier in the two-stage approach, and single-stage classifier based on neural network with 40 neurons hidden layer). Indeed, the proposed approach provided good global performance rates (sensitivity = 73.3%, specificity = 94.6%). In addition, in terms of computational requirements, it was sufficiently efficient for possible smartphone-based image analysis applications.

In 2019, Wang et al. performed a new study, somewhat following that in 2017 [45], aimed at developing a DFU recognition system able to determine the wound boundary in images acquired under different conditions [46]. In particular, the analyzed variations were those in illumination and viewing angles. In addition, images may have contained background objects, different from wounds, in the proximity of the wound boundary. Two image datasets were analyzed. The first dataset was composed of images of moulage wounds placed on an artificial foot. A total of 162 images of six moulage wounds were collected (27 images for each wound, at three different scales, three different viewing angles, and three different illumination conditions). The second dataset consisted of 100 images of actual DFU from 15 subjects followed over a two-year period (i.e., the dataset used in the 2017 study [45]). To determine the wound area, an Associative Hierarchical Random Field (AHRF) model was proposed. This model can be viewed as an extension of the Conditional Random Field (CRF) model, which is a discriminative machine learning approach that directly models the conditional probability of different class labels (such as wound and no-wound), given a set of images. In fact, it was claimed that AHRF has advantages compared to traditional CRF, as the former allows the use of image features defined at any scale and captured under different lighting conditions, and it requires no human intervention (wound determination is completely automatic). Following comparison of the AHRF model to some traditional CRF models, better overall performance was found for the former (specificity: >95% and sensitivity: >77%). With regard to the possible comparison to deep learning models, specific tests were not performed, but it was hypothesized that, when working with a small number of DFU images, AHRF is likely to outperform deep learning. On the other hand, when the number of images increases, the situation probably inverts, since the AHRF performance likely reaches a plateau, whereas deep learning has much more trainable parameters as compared to AHRF, typically allowing to improve performance.

In the same year, Ohura et al. assessed the possibility for segmentation of DFUs (as well as venous leg ulcers) by a CNN being trained using pressure ulcer (PU) images [47]. To this end, a dataset was exploited with 400 PU images and 20 DFU images. Different CNN architectures were analyzed, specifically SegNet, LinkNet, U-Net and U-Net with the VGG16 encoder pre-trained on the ImageNet dataset (U-Net_VGG16). It was found that the best results were achieved through U-Net, being the best compromise between performance (showing high specificity (0.943) and sensitivity (0.993)) and computational time. Other considered performance metrics included accuracy, AUC_ROC_, MCC and DICE. It was concluded that the proposed approach may be adequate for practical wound assessment of DFUs in eHealth applications.

One 2019 study by Goyal et al. focused on the implementation of an algorithm for automatic, real-time localization of DFU [48]. Specifically, conventional machine learning and deep learning techniques were implemented. As regards the conventional machine learning implementation, a SVM model with quadratic polynomial kernel was initially generated, but it was subsequently discarded for real-time applications because of its slowness in solving the DFU localization task. As regards the deep learning implementation, several CNNs were generated, characterized by different hyperparameter settings and different object localization meta-architectures (namely, Region-based Convolutional Neural Networks (R-CNN), Region-based Fully Convolutional Networks (R-FCN) and Single Shot Multibox Detector (SSD)). The two-tier transfer learning technique was applied, which exploits the implementation of networks pre-trained on massive datasets of non-medical images, thus avoiding the possibility that the networks are generated only from the limited medical images available. Two experts identified the ROIs in the dataset, consisting of 1775 DFU images. These ROIs were used as ground truth for the performance evaluation. In detail, the metrics considered for performance evaluation were speed, size of the model, mean average, precision and overlap percentage. It was found that all deep learning methods were characterized by good localization capabilities of multiple DFUs, with high inference rate. The SSD meta-architecture obtained the best inference speed, while the R-CNN generated the most accurate results (especially assessed by mean Average Precision (mAP)). This study showed that deep learning models are capable of real-time DFU localization, although they should be further enhanced using more extensive datasets. In 2020, Goyal et al. published a similar study, in that case focused on a specific CNN (called DFUNet), whose performance was compared to that of traditional networks, such as AlexNet, GoogLeNet, LeNet [49].

Again in 2020, another work by Goyal et al. focused on the implementation of a model for the detection of ischemia and infection in DFUs [50]. The first step consisted of data augmentation of the initial dataset comprising 1459 DFU images, carried out with a technique based on a deep DFU localization algorithm. Secondly, information about the colors of the ROIs was extracted from the DFU images, to identify the visual cues important for the detection of ischemia/infection. This step was implemented by a new method proposed by the authors, called Superpixel Color Descriptors (SPCD). Finally, several algorithms were implemented for ischemia/infection classification. From the context of machine learning, RF, Bayesian Network (BN) and MLP models were proposed, along with CNN algorithms (i.e., InceptionV3, ResNet50, and InceptionResNetV2). Finally, an ensemble CNN approach was employed, which combined the features of the three CNN models and used a SVM to perform the classification. Two experts identified the presence of ischemia/infection in the images of the dataset, and their assessments were used as the ground truth for classifier training. Traditional classification metrics were considered for performance evaluation (accuracy, precision, sensitivity, specificity, F1-score, MCC, AUC_ROC_). Based on those metrics, it was concluded that the ensemble CNN algorithm performed best in both the ischemia classification (90% accuracy) and the infection classification problem (73% accuracy). In general, better results were obtained in the ischemia detection rather than in the infection detection problem (average accuracy of 83.3% vs. 65.8%).

In the same year, Kim et al. performed a study aimed at predicting the healing of DFU, using both clinical features extracted from patients’ electronic health records (EHRs) and image features extracted from photographs (simply taken by a smartphone or tablet camera) [51]. A wide set of clinical features (48 variables) were considered, as derived from the EHRs of 2291 visits over a three-year period, for 381 DFUs from 155 patients. Due to the large number of clinical features, some missing values were present, but the problem was overcome by the imputation of each missing value as the Euclidean distance-weighted mean of the three most similar data samples (according to the not-missing features), using the KNN algorithm. The DFU photographs were manually segmented, then processed to extract color and texture features. In addition, deep learning-based features were extracted from the global average pooling (GAP) layer of the ResNet50, which is a 50-layer-deep CNN, with a pre-trained version available based on training over the wide ImageNet dataset. RF and SVM models were then trained for the prediction of eventual wound healing. Somewhat surprisingly, the models built with hand-crafted imaging features alone outperformed models built with clinical or deep learning features alone. Models trained with all features performed similarly against models trained with hand-crafted imaging features (performance metrics were AUC_ROC_, accuracy, precision, recall, and F1-score). It was concluded that since the most important features are predominantly hand-crafted imaging features, one application for predicting prognosis of DFUs may not require relevant computational resources (as it often happens for machine learning/deep learning approaches), and hence it could be adequate for running in a smartphone environment.

In 2021, the study by Al-Garaawi et al. aimed at developing a method for DFU classification through the use of CNN, in which texture information on the DFU is used as the model input in addition to a RGB image of the ROI [52]. In particular, the ability to discriminate between healthy subjects and subjects affected by DFU, as well as between ischemia and non-ischemia and infection and non-infection, was evaluated. Different model inputs were employed, starting from a single input based on the RGB image alone (this being the reference approach, used for comparison with other approaches based on multiple inputs). Thereafter, the RGB image complemented with an image containing texture information was considered as a model input. Texture information was obtained from the RGB image using the local binary patterns (LBP) technique of texture description: several LBP responses were extracted and merged with the RGB image to obtain an image with improved textures. The final CNN model was obtained by gradually adjusting the parameters such as the number of convolutions and max-pooling, and the number of filters to yield the final texture-enhanced image. The model was trained using the binary cross-entropy loss function, optimized with adaptive moment estimation. Performances, expressed in terms of sensitivity, specificity, precision, accuracy, F1-score, and AUC_ROC_, were compared with the results obtained by the state-of-the-art algorithms AlexNet and GoogLeNet on the same dataset. It was concluded that the CNN classification model with the texture-enhanced image as input outperformed the state-of-the-art methods, obtaining higher AUC_ROC_ values for the classification of DFU, as well as for ischemia and infection.

The article by Yap et al. is somewhat different from the others included in this review study [53]. In fact, Yap’s article reports the results of a challenge, called DFUC2020, in which the aim was to train deep learning algorithms for DFU detection on a dataset composed of 4000 images (50% used for training, 50% used for testing). The algorithms that obtained the best performances in terms of different metrics, such as F1-score, mAP, true and false positives, were summarized: R-CNN, three variants of R-CNN, an ensemble method, two versions of You-Only-Look-Once (YOLO), EfficientDet, and a Cascade Attention Network. In all implementations, to increase the number of images for training the algorithms, different data augmentation techniques were employed. In addition, a post-processing phase was implemented for each model to mainly minimize the number of false positives, though it was noted that this would lead to increased healthcare costs if these algorithms were to be used in clinical practice. Four images were generated through data augmentation from each image, and the predictions obtained on these derived images were combined to obtain the final prediction on the original image. Predictions generated by the R-CNN model and its variations were combined in post-processing with the ensemble Weighted Boxes Fusion algorithm to generate an averaged localization of the predictions (i.e., the region of each image where the algorithm identified the presence of the DFU, if any). As mentioned above, two versions of the YOLO real-time object detection model were implemented for the challenge: YOLOv3 and YOLOv5. Generally, the YOLO approach transforms the object detection problem into a regression problem. Specifically, YOLOv3 predicts bounding boxes (i.e., the regions with DFU presence) on different scales, and a score from LR is associated with each generated bounding box. YOLOv5 focuses on the exploitation of data augmentation techniques. The EfficientDet model was based on a feature fusion technique that merged the detection of image regions at various resolutions. The Cascade Attention Network was composed of a series of neural networks. Overall, the best approach was considered one of the R-CNN variants (a Faster R-CNN called Deformable Convolution model), which obtained the best results in terms of mAP (0.6940) and F1-score (0.7434). On the other hand, based on the significant number of false positives obtained during testing by all analyzed algorithms, it was concluded that further research is required for improved DFU detection. It has to be noted that in the same period, another article, by Cassidy et al., presented some details about the DFUC2020 dataset, including the indication of assessment methods and benchmark algorithms available, and results of an initial evaluation [54].

The study by Xu et al. was again based on the consideration that identifying the presence of infection and ischemia in DFU is important for treatment planning [55], as in the 2020 study by Goyal et al. [50]. It was claimed that the deep learning-based classification of infection has shown promising performance, especially when using deep neural networks (CNNs in particular) to extract discriminative features from DFU images and predict class probabilities (specifically, infection and/or ischemia presence/no presence). However, it was observed that in the typical CNN-based methods, in the testing phase the classification depends on the individual input image and trained networks parameters, but the knowledge provided in the training data is not explicitly (fully) exploited. To better use the training data knowledge, the Class Knowledge Banks (CKBs) approach was proposed. In this approach, each unit in a CKB is used to compute similarity with an input image. The averaged similarity between units in the CKB is a representative parameter of the input image that can be helpful in the image classification, since the classification depends not only on the image and the trained parameters, but also on the class knowledge extracted from the training data and stored in the CKBs. The proposed approach was applied to the same dataset used by Goyal et al. in the 2020 study [50]. The dataset included 628 non-infection and 831 infection cases, as well as 1249 non-ischemia and 210 ischemia cases. Due to the obvious class imbalance issue (especially for ischemia/non-ischemia classification), data augmentation strategy was applied, this ending in 9870 image patches for ischemia/non-ischemia, and 5892 for infection/non-infection. Considered performance metrics were accuracy, sensitivity, precision, specificity, F1-score, AUC_ROC_.

The study by Viswanathan et al. aimed to evaluate the accuracy of an imaging device working on multispectral autofluorescence (Illuminate^®^, Adiuvo Diagnostics, Private Limited, Chennai, India) in detecting bacterial Gram type compared with standard culture methods [56]. Indeed, evidence-based assessment of early infections can be of help in providing the right first-line treatment, thus improving the wound healing rate. A total of 178 patients with DFU were recruited, and 203 tissue samples were taken from the wound color-coded device images of the infected regions, as indicated by the device artificial intelligence algorithm. The device capability in classifying the right Gram type (positive or negative), or no-infection condition, was expressed in terms of accuracy, PPV and NPV. The NPV for no-infection reached 96%, whereas PPV and NPV for Gram-positive or Gram-negative ranged from 80% to 92%. Unfortunately, no details were provided on the artificial intelligence algorithm of the device.

In 2022, similarly to previous studies [50,55], Güley et al. carried out a study aimed again at addressing the “classic” four-class classification problem for DFU [57], with the four classes being (i) infection, (ii) ischemia, (iii) both infection and ischemia, and iv) control case (i.e., neither infection nor ischemia). Indeed, identification of infection and/or ischemia in a wound is important, since the presence of these conditions can significantly prolong treatment and often results in limb amputation, with more severe cases resulting in terminal illness. For the study purposes, the DFU Challenge 2021 (DFUC2021) dataset was exploited, including 15,863 images for which ground truth labels were available for the indicated four classes. As regards the machine learning approach, the study relied on the Generally Nuanced Deep Learning Framework (GaNDLF). In fact, GaNDLF facilitated the model development by providing tools to rapidly incorporate techniques such as cross-validation, data pre-processing, and data augmentation. A series of VGG architectures were evaluated, with different layers, training strategies, and data pre-processing and augmentation techniques. One of the advantages of the VGG architectures for the study’s aims was the use of small convolutional filters and spatial padding, with the goal of preserving the original resolution of the input images. Three versions of the VGG architecture were trained, namely the VGG11, VGG16, and VGG19. For transparency, all implementations were made available through the GaNDLF. When compared to the other models in DFUC2021, the best model in this study ranked in the 2nd–7th range, depending on the performance metrics considered (AUC_ROC_, F1-score, recall).

Wang et al. aimed to analyze the ability of magnetic resonance imaging (MRI) in assessing the effectiveness of DFU treatment [58]. A group of 78 patients with DFU were randomly split into the experimental group, treated with a composite skin graft, and the control group, treated with an autologous skin graft (39 patients in each group). MRI scans were performed before and after treatment. A deep learning algorithm model, called single shot detector (SSD), was applied to the MRI images to locate and extract features of the foot wounds requiring skin grafting. SSD exploits a CNN to detect all objects of interest within an image in one pass, at difference with a sliding window approach. It was found that in the experimental group, some parameters of the MRI images (signal intensity parameters) differed pre- and post-intervention. As regards specific clinical parameters of the therapeutic effect, it was also found that the wound healing time and complete healing rate were not different between experimental and control groups, but the wound recurrence rate and the scar status (rated with a specific score) were considerably decreased in the former. Interestingly, it was reported that MRI-derived signal intensity parameters correlated with the therapeutic effect of the intervention, as expressed by the previously indicated clinical parameters. The considered performance metrics were accuracy, specificity, sensitivity, and AUC_ROC_.

In the study by Yogapriya et al., clinical signs and symptoms of local inflammation were used to diagnose diabetic foot infection, in the hypothesis that infections have significant implications in predicting the likelihood of amputation in DFU [59]. The analyzed dataset consisted of 5890 DFU images, with 2945 images for infection and 2945 images for non-infection. The dataset underwent proper data augmentation approach, ending with 29,450 images. Then, a CNN, named Diabetic Foot Infection Network (DFINET), was developed from the DFU images to predict infection or non-infection, based on 22-layer CNN architecture, with one normalization layer and one dropout layer. The DFINET hyperparameters were analyzed and fine-tuned to enhance the model performance. DFINET was also compared to other models, such as AlexNet, GoogLeNet, VGG16. Several performance metrics were computed (accuracy, sensitivity, specificity, precision, F1-score, MCC, PPV, and NPV). Interestingly, DFINET outperformed all other models in almost all metrics. DFINET accuracy reached 91.98%, being much higher than that of the other models, not exceeding 83%.

Chan et al. aimed to validate an artificial intelligence-enabled wound imaging mobile application (CARES4WOUNDS system, Tetsuyu, Singapore) against traditional wound assessment measurements as performed by a trained specialist nurse in patients with DFU [60]. Seventy-five wound episodes (median wound area of 3.75 cm^2^) were collected from 28 diabetic patients, and a set of 547 wound images were analyzed. Excellent intra-rater reliability of CARES4WOUNDS was observed, by analysis of three different images of the same wound (0.933–0.994 range). Most importantly, between CARES4WOUNDS assessment and nurse measurement there was also good inter-rater reliability for wound length, width, and area (0.825–0.934 range). Unfortunately, no details were reported about the artificial intelligence algorithms used by the CARES4WOUNDS system.

## 4. Discussion

### 4.1. Introductory Comments and Comparison with Previous Review Studies

In this review, we focused on the use of artificial intelligence techniques applied to data derived from different sensors and technologies for the study of diabetic foot syndrome. Previous reviews, such as some of those mentioned previously [17,19], indicated diabetic foot as one of the fields for the application of artificial intelligence in the more general context of diabetes, but they were not focused specifically on the diabetic foot. On the other hand, one review focused on the diabetic foot, but artificial intelligence methodologies were only covered in a small part of the review [61]. Another review study focused on artificial intelligence in diabetic foot [62], but it did not report details either on the physiological/clinical study outcomes or on the specific artificial intelligence techniques used in each of the analyzed studies. In contrast, another recent review study presented with care and in detail some artificial intelligence-based studies on the diabetic foot [63], but the addressed studies were limited to those based on diabetic foot imaging and analyzed with deep learning methodologies. As shown by our review study, imaging is a relevant aspect in diabetic foot investigation, since two-thirds of the revised studies focus on, or at least included, some image data. On the other hand, one-third of the studies in our review did not include imaging at all. It is also worth noting that not all studies with imaging exploited deep learning approaches, since some studies applied other artificial intelligence algorithms. In summary, to our knowledge, this is the first review study analyzing a vast set of research studies, where the diabetic foot was investigated with a wide bunch of heterogeneous approaches for data collection and artificial intelligence-based analysis.

### 4.2. Comments on the Specific Sections Summarizing the Studies Pertinent for The Review

In more detail, our review stratified the relevant studies into four sections (namely, Section 3.1.1, Section 3.1.2, Section 3.2.1 and Section 3.2.2). The first of these sections relates to the screening for diabetic foot syndrome and risk prediction for ulceration from socioeconomic and sociodemographic data, as well as clinical data, but without exploitation of any imaging of the foot. What appears somewhat striking is the remarkable heterogeneity in the number of subjects involved in the different studies, ranging from a few dozen to some millions. On the other hand, it has to be acknowledged that, typically, a lower number of patients relates to more accurate patient phenotyping, i.e., a higher number of collected parameters and measured variables, and hence a higher number of features considered in the artificial intelligence-based approaches. From this point of view, it is worth noting the heterogeneity in such approaches, though some algorithms were somewhat more commonly used (Support Vector Machine, K-Nearest Neighbor, Naïve Bayes, and Random Forest). Finally, an interesting aspect of the studies falling in this section is that most of them (six out of seven) were published in the last two years (from 2020 onwards). In our opinion, this is an indication of the remarkably growing interest in early diabetic foot screening based on possibly simple variables and parameters, not necessarily requiring imaging or other somewhat complex health technologies. We will comment on this aspect in one of the next sections of the Discussion.

The second section of this review addressed the studies about diabetic foot syndrome screening and ulceration risk prediction where imaging technologies have been exploited (mainly, though not exclusively, thermal imaging of the plantar foot). Compared to the studies falling in the previous section, the studies in this section are more homogeneous in terms of the number of subjects, which typically is not particularly high (not exceeding a few hundred subjects). This may be reasonable considering that studies including imaging are more complex (and likely expensive) than studies only relying on simpler variables and parameters, but on the other hand, one may wonder whether the use of imaging is in fact cost-effective for screening purposes (which, by definition, should be ideally applied to large cohorts of subjects). However, it has to be acknowledged that similarly to the studies in the previous section, the great majority of studies in this section are again very recent (9 out of 10 studies since 2020), suggesting that the scientific community typically considers imaging-based approaches appropriate for screening, despite the potential limitations in terms of complexity and costs if applied to large populations. It also has to be noted that some studies complemented imaging information with other data (socioeconomic/demographic and/or clinical), thus ending with heterogeneous features to be handled. With regard to the type of artificial intelligence approach, neural networks (typically convolutional, possibly deep networks) have been often exploited in the studies of this section, thus being more homogeneous than the studies in the previous section. It is likely that neural networks have been found to be efficient in dealing not only with imaging-derived features, but also with the heterogeneous features that are present in these studies (as noted above), and this is reasonable in light of their flexibility. On the other hand, it has to be considered that the results derived by neural networks, especially the most complex (deep networks), are typically difficult to explain. Specifically, since such networks include several interconnected processing nodes, it is often difficult to understand how the node weights result in the predicted network output. For this reason, neural networks may not easily be accepted by users without specific expertise in this type of algorithms, and thus from this point of view the neural network approach may not be the easiest one to integrate into clinical practice. On the other hand, it is worth noting that the issue of neural networks’ interpretability is being addressed in some studies [64,65,66].

The following (third and fourth) sections of the review focus on the detection and grading of already overt ulcerations, and on related prognosis and care. In the third section, as in the first section, we analyzed studies with no exploitation of imaging techniques. The number of subjects in these studies again does not exceed a few hundred, but this is reasonable considering that patients with overt wounds are clearly less frequent than subjects screened for wounds (addressed in the first two review sections). Features considered in this section are heterogeneous, including data directly related to the ulcer (ulcer area, and/or markers of ulcer type and severity), as well as other demographic and clinical data (especially, patient’s medical history). As regards the artificial intelligence approaches, there was heterogeneity among studies, although some algorithms were more commonly used (Support Vector Machine, Extreme Gradient Boosting). The majority of studies were again very recent (from 2020 onwards).

In the last (fourth) section, we analyzed studies about already overt ulceration with exploitation of imaging techniques, with “traditional” (visible light) imaging being the most common approach (also derived by non-professional devices, such as smartphones). The number of patients in these studies was similar to that in the studies in the third section (a few hundred at most, when indicated), but the overall number of images in some studies was remarkable (up to more than 10,000 images). On the other hand, interestingly, the image-derived features were rarely complemented by other types of data, likely indicating the general investigators’ belief that other data are not expected to add relevant information to those provided by actual images of the wound, especially if several images are available. In terms of algorithms, similarly to the second section of this review (dealing with images as well), neural networks (especially convolutional, in different variants) were the most commonly used. As regards publication time, again the majority of studies were published after 2020, but here a not negligible number of studies were published earlier (though still recently). This may indicate that the investigation of overt diabetic foot ulcers by imaging was the first category of studies attracting considerable interest, within the context of artificial intelligence applications in the diabetic foot.

### 4.3. Other Comments and Our Personal View for Future Studies in the Field of the Diabetic Foot

What are the main conclusions that can be drawn from the presented studies? As mentioned above, these studies were quite various in terms of both technologies and methodologies, as well as with regard to the main study outcomes. In particular, some studies were mainly devoted to diabetic foot ulcer prevention, whereas others were focused on the prognosis and care of already overt ulcers. In our opinion, this heterogeneity indicates on one the hand that it is still not clearly established as to what artificial intelligence methodologies are more appropriate in diabetic foot issues, and this calls for further research. On the other hand, the different and various physiological and clinical outcomes of the examined studies suggest that artificial intelligence may be useful for several purposes, within the general context of the research activity in diabetic foot syndrome.

Some of the analyzed studies were aimed at the early identification of the risk for the onset of diabetic foot syndrome, and especially for the prevention of diabetic foot ulcers (see Table 1 and Table 2). In these studies, the artificial intelligence methodologies were typically exploited to identify, among a wide battery of variables, the main predictors of diabetes foot risk. Some of the examined studies analyzed potential diabetes foot predictors derived from general patients’ clinical records, or using socioeconomic/sociodemographic information. When direct quantitative measures of the foot health condition was added, this was typically limited to the foot temperature, considered in terms of thermal images (thermograms). Foot temperature is certainly an appropriate variable to be measured for basic early screening of the diabetic foot, and measurement can even be performed at home through a traditional digital thermometer [67,68,69,70,71,72]; of note, studies also suggested thresholds in terms of temperature variation between the two feet, which should trigger more accurate examinations, in cases where the threshold is exceeded [73,74]. However, in our opinion, other simple quantitative measures may be performed for a first-level early screening of the foot condition, possibly even at home if implemented in appropriate devices. First, we suggest the measurement of foot skin resistance. Indeed, it has been reported that skin resistance can be a marker of endothelial damage [75], and this of course appears important in diabetic foot syndrome. Another relevant simple measure may be the degree of humidity in the foot skin [76,77]. Furthermore, plantar pressure measurement appears relevant as well [78,79,80]. Notably, special insoles have been proposed that integrate several pressure sensors, and thus are able to monitor the pressure exerted by the foot at different locations of the plantar region. As an example, the Parotec insoles system includes 16 pressure sensors, distributed among the hallux, lateral heel, lateral midfoot, medial heel, medial midfoot, and first, second to third, and fourth to fifth metatarsal head regions (thus, for a total of eight regions, with two sensors each) [81,82]. Another device is the F-Scan insole system, which detects foot pressure by means of ultra-thin resistive film sensors distributed over the whole plantar region [83]. Interestingly, the system offers the opportunity of using sensor tabs of different sizes and for different types of boot (ski, military, work boots, etc.), thus essentially providing solutions tailored for the specific user needs [84]. In another study, three different insole systems were analyzed and compared, one of which being the F-Scan system, and the others being the Medilogic system and the Pedar system [85]. The study findings were that the F-Scan and Medilogic systems perform better for pressure values in the 200–300 kPa range, whereas Pedar essentially performs satisfactorily across all pressure values, thus suggesting its validity for use in both clinical and research settings [85]. Other insole systems proposed particular pressure sensing solutions, based on optical fiber technology (the so-called fiber Bragg grating sensors) [86,87,88]. Of note, some studies emphasized the opportunity to produce such pressure measurement insoles at low cost, in the order of USD 50 for all insole components [89].

Thus, skin temperature, resistance, humidity and plantar pressure may provide important and somewhat complementary information about the foot condition. In addition, since small and inexpensive sensors are available for such measures, they may be integrated into a single device adequate for personal, at-home use (possibly, a “special” scales device, with a shape similar to traditional body weight scales, or even an in-shoe device). It is worth noting that some studies already moved in the direction of integrating different sensors for diabetic foot monitoring. Specifically, some recent studies the proposed integration of temperature and pressure sensing, plus, possibly, glucose levels in sweat [90,91]. However, as stated above, skin resistance and humidity sensors should be integrated; we suggest that there should be at least two resistance sensors for each foot, one possibly placed between the second and third metatarsal head regions, and one between the lateral and medial heel regions, whereas at least one humidity sensor per foot should be used, possibly placed between the lateral and medial midfoot regions.

All these quantitative measures (thus, not only temperature) could then be exploited by artificial intelligence approaches, possibly in addition to other clinical variables by patient’s clinical records and the socioeconomic/sociodemographic variables. This would likely allow improvement in the performance of the artificial intelligence models for the prediction of the risk for diabetic foot syndrome (or, specifically, for diabetic foot ulceration), without the need for more complex and expensive technologies (such as imaging). Of note, in our opinion, despite some pitfalls (as discussed above), neural networks may be a very valuable option among all those available in the artificial intelligence context, for the neural networks ability to “learn” and hence possibly optimize the diabetic foot strategy for different categories of diabetic patients, and hopefully for a single patient. This appears consistent with the expected future for the care of diabetes (thus including diabetic foot care), moving towards precision and even personalized (i.e., for the single individual) medicine [92,93,94,95,96]. In fact, the integration of different sensing strategies, which is advisable for the reasons explained (mainly, the heterogeneity of diabetic foot syndrome), coupled with appropriate artificial intelligence approaches (able to “evolve” and improve their performances), offers important grounds for the application of concepts in the context of precision and personalized medicine.

## 5. Conclusions

In conclusion, we analyzed studies that applied several artificial intelligence methodologies for the analysis of different measured data or relevant information related to diabetic foot syndrome. Given the complex, multifactorial nature of this syndrome, artificial intelligence appears to be an appropriate approach to identify the variables of relevance for prescribed scientific purposes, among many variables possibly involved. The analyzed studies showed promising results achieved by the employed artificial intelligence techniques. However, with regard to the specific aspect of diabetic foot early screening, in our opinion, future studies may benefit from the integration of further quantitative measures of the foot condition, which may be obtained by simple and inexpensive sensors.

## Figures and Tables

**Table 1:** Summary information related to the studies concerning diabetic foot syndrome screening—studies based on socioeconomic and sociodemographic data, and on different measured clinical data, but without imaging. The list of abbreviations is reported in the Abbreviations section.

Reference	Aim	Population	Measured/Collected Data	AI Methods	Metrics
Singh et al., 2013 [21]	Finding DFU risk associated with 5 SNPs in the TLR4 gene	255 T2DM patients (125 with DFU, 130 without DFU)	Genomic DNA, clinical and laboratory evaluation, family history, habits, duration of disease	ANN	Accuracy
Ferreira et al., 2020 [22]	Early identification of T2DM patients at high risk of developing DFU	239 T2DM patients	Health conditions, changes perceived in feet, information on foot care, type of footwear, socioeconomic and sociodemographic conditions	CNL	Accuracy, sensitivity, specificity
Schäfer et al., 2020 [23]	Risk of DFU development/amputation in diabetic people	246,705 diabetic patients	Patient’s health and socioeconomic data	LR, RF	Accuracy, AUC_ROC_
Stefanopoulos et al., 2021 [24]	Prediction of DFU	Over 10 million diabetic patients, 326,853 of which with DFU	Nationwide Inpatient Sample dataset (2008–2014, USA)	CTREE	Accuracy, sensitivity, specificity, AUC_ROC_
Haque et al., 2022 [25]	Prediction of diabetic neuropathy or overt DFU	21 subjects (6 with diabetic neuropathy, 9 with DFU, 6 controls)	Electromyography and ground reaction forces	DA, EC, KC, KNN, LC, NB, SVM, BDC	Accuracy, sensitivity, precision, AUC_ROC_, F1-score
Nanda et al., 2022 [26]	Detection of DFU risk and of its severity (according to Wagner Score)	160 T2DM patients (80 with DFU, 80 without DFU)	Clinical and biochemical risk factors for DFU	SVM, NB, KNN, RF, ensemble learners; Relieff, Info Gain, Gain Ratio and Chi-squared (for feature ranking)	AUC_ROC_, F1-score, MCC
Troitskaya et al., 2022 [27]	Prediction of onset of diabetic foot syndrome	198 diabetic patients without complications, and 199 diabetic patients with signs of diabetic foot	Polymorphisms of genes, markers of endothelial dysfunction	MLP	Accuracy, sensitivity, specificity, AUC_ROC_

**Table 2:** Summary information related to the studies concerning diabetic foot syndrome screening—studies based on imaging. The list of abbreviations is reported in the Abbreviations section.

Reference	Aim	Population	Measured/Collected Data	AI Methods	Metrics
Toledo Peral et al., 2018 [28]	Identification and classification of skin macules	19 diabetic patients (without DFU)	82 photographs of skin macules	ANN	Accuracy, confusion matrix
Cruz-Vega et al., 2020 [29]	Classification of diabetic foot thermograms (five classes)	Diabetic patients (number not specified)	110 thermograms	MLP, SVM, CNN (GoogLeNet and AlexNet, and new CNN: DFTNet)	Accuracy, sensitivity, specificity, precision, AUC_ROC_, F1-score
Khandakar et al., 2021 [30]	Classification in diabetic or control subject for early detection of DFU risk	122 diabetic and 45 control subjects	Gender, age, weight, height, pairs of thermograms	Machine learning algorithms on features extracted from images; deep CNN algorithms on images	Accuracy, sensitivity, specificity, precision, AUC_ROC_, F1-score
Arteaga-Marrero et al., 2021 [31]	Proof-of-concept of foot sole segmentation of multimodal images	37 healthy subjects	74 visual-light, infrared and depth images	CNN (U-Net), deep CNN (SegNet)	Accuracy, sensitivity, specificity, precision, DICE, spatial overlap
Dremin et al., 2021 [32]	Identification of skin differences between diabetic and healthy subjects	32 healthy subjects (1st study phase), 20 diabetic and 20 healthy subjects (2nd study phase)	Photonic data (hyperspectral imaging and parameters)	ANN (MLP)	Accuracy, sensitivity, specificity, AUC_ROC_
Khandakar et al., 2022 (two articles) [33,34]	Early detection of DFU risk, clustering of severity in foot temperature anomalies	122 diabetic and 45 control subjects	Gender, age, weight, height, pairs of thermograms	Machine learning algorithms; deep CNN algorithms; K-mean clustering	Accuracy, sensitivity, specificity, precision, AUC_ROC_, F1-score
Zhang et al., 2022 [35]	Detection of DFU risk and of its severity (according to Wagner Score)	203 diabetic patients	Sociodemographic and clinical data, and CTA images	ANN, with MLP algorithm	Accuracy, PPV, NPV, sensitivity, specificity, AUC_ROC_
Bouallal et al., 2022 [36]	Segmentation of diabetic foot	145 diabetic and 54 healthy subjects	398 pairs of thermal and RGB images	DE-ResUnet	IoU, Acc
Muralidhara et al., 2022 [37]	Detection of DFU risk and of its severity (6 classes)	122 diabetic and 45 control subjects	Thermograms	CNN algorithm coupled with class balancing (weighted classification and data augmentation)	Accuracy, sensitivity, specificity, precision, F1-score

**Table 3:** Summary information related to the studies about overt diabetic foot ulcers—studies based on socioeconomic and sociodemographic data, and on different measured clinical data, but without imaging. The list of abbreviations is reported in the Abbreviations section.

Reference	Aim	Population	Measured/Collected Data	AI Methods	Metrics
Yusuf et al., 2015 [38]	Validation of e-nose in detection of bacteria responsible for DFU infection	Patients with DFU (number not specified)	In vitro bacteria samples	SVM, KNN, LDA, PNN	Accuracy, sensitivity, specificity, precision
Huang et al., 2018 [39]	Quantification of rehabilitative efficiency of Buerger’s exercise; discrimination between healthy and diabetic subjects	30 diabetic and 15 healthy subjects	Tissue oxygen saturation in lower limbs and relative total hemoglobin concentration	RBFNN	F1-score
Lin et al., 2020 [40]	Prediction of amputation/mortality in patients with DFU	200 patients with DFU	Biochemical markers, clinical data and presence of complications	Cox regression, BPNN (also with GA)	Sensitivity, specificity, AUC_ROC_
Du et al., 2021 [41]	Prediction of amputation/mortality in inpatient with DFU before/after pandemic	23 inpatients with DFU	Clinical and laboratory data, WIFI classification	LR, SVM, RF, GBDT, ANN, XGBoost	Accuracy, NPV, PPV, sensitivity, specificity, AUC_ROC_
Xie et al., 2022 [42]	Prediction of in-hospital amputation	618 patients with DFU	Demographic features, medical and medication history, clinical and laboratory data, Wagner and WIFI classifications	LightGBM	Accuracy, NPV, PPV, sensitivity, specificity, AUC_ROC_
Margolis et al., 2022 [43]	Prediction of wound healing	204 patients with DFU	Wound area, duration, depth, site, arterial flow, BMI, history of dialysis	LR, LASSO	AUC_ROC_
Deng et al., 2022 [44]	Prediction of mortality in DFU+HCE patients	27 inpatients with DFU+HCE, 93 inpatients with isolated DFU	HCE presence, mortality occurrence, clinical data	XGBoost	AUC_ROC_, accuracy, sensitivity, specificity

**Table 4:** Summary information related to the studies about overt diabetic foot ulcers—studies based on imaging. List of abbreviations is reported in the Abbreviations section.

Reference	Aim	Population	Measured/Collected Data	AI Methods	Metrics
Wang et al., 2017 [45]	Detection of DFU area	15 patients with DFU	100 DFU images	Two-stage SVM	Sensitivity, specificity
Wang et al., 2019 [46]	Automatic DFU localization under different conditions	15 patients with DFU	162 moulage wound images + 100 actual DFU images	AHRF	Sensitivity, specificity
Ohura et al., 2019 [47]	Automatic DFU localization	Patients with DFU (number not specified)	400 pressure ulcer images and 20 DFU images	SegNet, LinkNet, U-Net and U-Net with VGG16	Accuracy, sensitivity, specificity, AUC_ROC_, MCC, DICE
Goyal et al., 2019 (and 2020) [48,49]	Real time automatic DFU localization	Patients with DFU (number not specified)	(Up to) 1775 DFU images	From machine learning: SVM; from deep learning: R-CNN, R-FCN, SSD; DFUNet	mAP, overlap percentage, size of model, speed; accuracy, sensitivity, specificity, precision, AUC_ROC_, F1-score
Goyal et al., 2020 [50]	Detection of ischemia/infection in DFU	Patients with DFU (number not specified)	1459 DFU images	From machine learning: RF, BN, MLP; from deep learning: three CNN (InceptionV3, ResNet50, and InceptionResNetV2), ensemble CNN based on the three CNN	Accuracy, sensitivity, specificity, precision, AUC_ROC_, F1-score, MCC
Kim et al., 2020 [51]	Prediction of DFU prognosis	155 patients with 2291 visits for 381 DFUs	Clinical variables, smartphone-based photographs	ResNet50, RF, SVM	Accuracy, precision, recall, AUC_ROC_ F1-score
Al-Garaawi et al., 2021 [52]	DFU classification, detection of ischemia, detection of infection	Patients with DFU (number not specified)	RGB images and derived information about texture of the ROI	CNN	Accuracy, sensitivity, specificity, precision, AUC_ROC_, F1-score
Yap et al., 2021 (and Cassidy et al., 2021) [53,54]	DFU detection	Patients with DFU (number not specified)	4000 DFU images with expert annotations	R–CNN, three variants of R–CNN, an ensemble method; YOLOv3, YOLOv5; efficientDet; Cascade Attention Network	Precision, recall, true and false positives, F1-score, mAP
Xu et al., 2021 [55]	Detection of ischemia/infection in DFU	Patients with DFU (number not specified)	1459 DFU images	CKBs	Accuracy, sensitivity, specificity, precision, AUC_ROC_, F1-score
Viswanathan et al., 2021 [56]	Identification of wound Gram type infections	178 patients with DFU, for 203 wound tissue samples	Autofluorescence images	Not specified	Not specified
Güley et al., 2022 [57]	Identification of wound infection and/or ischemia	Patients with DFU (number not specified)	15,863 DFU images (possibly with wound infection and/or ischemia)	VGG11, VGG16, VGG19	Recall, AUC_ROC_, F1-score
Wang et al., 2022 [58]	Ability of MRI images to describe therapeutic effect of skin grafting	78 patients with DFU (39 +39, for composite and autologous graft, respectively)	MRI images of DFU	Deep learning model (SSD)	Accuracy, sensitivity, specificity, AUC_ROC_
Yogapriya et al., 2022 [59]	Prediction of DFU non-infection or infection (risk for amputation)	Patients with DFU (number not specified)	5890 DFU images (2945 with foot infection, 2945 without infection)	CNN with normalization and dropout layers (DFINET)	Accuracy, NPV, PPV, sensitivity, specificity, precision, F1-score, MCC
Chan et al., 2022 [60]	DFU detection and measurement of its length, width, and area	Patients with DFU (number not specified)	547 DFU images	Not specified	Intra- and inter-rater reliability

## Data Availability

Not applicable.

## Abbreviations

This section reports the meaning of the abbreviations, in alphabetic order.

Acc	Accuracy per class
AdaBoost	Adaptive Boosting
AHRF	Associative Hierarchical Random Field
AI	Artificial Intelligence
ANN	Artificial Neural Network
Apo A1	Alipoprotein A1
AUC_ROC_	Area-Under-the-Curve of ROC curve
BDC	Binary Decision Classification
BMI	Body Mass Index
BN	Bayesian Network
BPNN	Back-Propagation Neural Network
CKBs	Class Knowledge Banks
CNL	Competitive Neural Layer
CNN	Convolutional Neural Network
CRF	Conditional Random Field
CTA	Computed Tomography Angiography
CTREE	Conditional Inference Tree
DA	Discriminant Analysis
DE-ResUnet	Double Encoder-ResUnet
DFINET	Diabetic Foot Infection Network
DFTNet	Diabetic Foot Thermograms Network
DFU	Diabetic Foot Ulcer
DFUC	Diabetic Foot Ulcer Consortium
DICE	Dice Similarity Coefficient
DT	Decision Tree
EC	Ensemble Classification
EHR	Electronic Health Record
EMG	Electromyography
GA	Genetic Algorithm
GaNDLF	Generally Nuanced Deep Learning Framework
GAP	Global Average Pooling
GBDT	Gradient Boosting Decision Tree
GBM	Gradient Boosting Machine
GRF	Ground Reaction Forces
HCE	Hyperglycemic Crisis Episode
IL-10	Interleukin-10
IoU	Intersection over Union
KC	Kernel Classification
KNN	K-Nearest Neighbor
LASSO	Least Absolute Shrinkage and Selection Operator
LBP	Local Binary Patterns
LC	Linear Classification
LDA	Linear Discriminant Analysis
LightGBM	Light Gradient Boosting Machine
LR	Logistic Regression
mAP	mean Average Precision
MCC	Matthews Correlation Coefficient
MLP	Multilayer Perceptron
MLR	Multivariate Linear Regression
MMP9	Metalloproteinase-9
MRI	Magnetic Resonance Imaging
NB	Naïve Bayes
NPV	Negative Predictive Value
PCA	Principal Component Analysis
PNN	Probability Neural Network
PPV	Positive Predictive Value
PU	Pressure Ulcer
RBF	Radial Basis Function
RBFNN	Radial Basis Function Neural Network
R-CNN	Region-based Convolutional Neural Network
RF	Random Forest
R-FCN	Region-based Fully Convolutional Networks
RGB	Red, Green, Blue
ROC	Receiver-Operating-Characteristic
ROI	Region of Interest
SHAP	Shapley Additive Explanations
SMOTE	Synthetic Minority Oversampling Technique
SNPs	Single Nucleotide Polymorphisms
SPCD	Superpixel Color Descriptors
SSD	Single Shot Detector
SVD	Singular-Value Decomposition
SVM	Support Vector Machine
T2DM	Type 2 Diabetes Mellitus
WIFI	Wound, Ischemia, Foot Infection
XGBoost	Extreme Gradient Boosting
YOLO	You-Only-Look-Once
